# Supplementary material for: Reverse Remodeling and Functional Improvement of Left Ventricle in Patients with Chronic Heart Failure Treated with Sacubitril/Valsartan: Comparison between Non-Ischemic and Ischemic Etiology
Source: J Clin Med. 2023 Jan 12;12(2):621. doi: 10.3390/jcm12020621 (PMC9864277; doi:10.3390/jcm12020621)
Supplement: Supplementary file 1 [file jcm-12-00621-s001.zip › jcm-2122979-supplementary.pdf]

Supplemental Materials

Supplementary Tables

Table S1. NYHA change at follow-up in all patients.

All	NYHA class at follow-up					
**NYHA class at baseline**	1	2	2.5	3	4	Total
2	7 (88)	**16 (36)**	2 (20)	0 (0)	1 (33)	26 (33)
3	1 (12)	28 (6)	8 (80)	**14 (100)**	2 (67)	53 (67)
Total	8	44	10	14	3	79 (100)

We report the results describing the NYHA functional class change following SV therapy. In the entire population of 79 patients, 27% of patients in NYHA II passed to NYHA I, 69.8% of patients in NYHA class III improve functional class

Legend: Bold: unchanged, green: improved, orange: worsened. Fisher's exact p value test =0.0002.

Table S2. NYHA change in follow-up in non-ischemic patients

Non-ischemic	NYHA class at follow-up					
**NYHA class at baseline**	1	2	2.5	3	4	Total
2	4 (100)	**11 (50)**	2 (33)	0 (0)	0 (0)	17 (46)
3	0 (0)	11 (50)	4 (67)	**4 (100)**	1 (100)	20 (54)
Total	4	22	6	4	1	37 (100)

In the subgroup of 37 non-ischemic patients, 23% of patients in NYHA II switched to NYHA I, 75% of patients in NYHA class III improve functional class

Legend: Bold: unchanged, green: improved, orange: worsened. Fisher's exact p value test=0.034.

Table S3. NYHA change in follow-up in ischemic patients.

Ischemic	NYHA class at follow-up					
**NYHA class at baseline**	1	2	2.5	3	4	Total
2	3 (75)	**5 (23)**	0 (0)	0 (0)	1 (50)	9 (21)
3	1 (25)	17 (77)	4 (100)	**10 (100)**	1 (50)	33 (79)
Total	4	22	4	10	2	42 (100.00)

In the subgroup of 42 ischemic patients, 33% of NYHA II patients switched to NYHA I, 66.6% of NYHA III patients improved functional class.

Legend: Bold: unchanged, green: improved, orange: worsened. Fisher's exact p value test=0.017.

Table S4. Change in diastolic dysfunction at follow-up in all patients.

All	Diastolic dysfunction at follow-up			
**Diastolic dysfunction at baseline**	1	2	3	Total
1	**21 (66)**	1(4)	0 (0)	22 (28)
2	9 (28)	**16 (64)**	4 (19)	29 (37)
3	2 (6)	8 (32)	**17 (81)**	27 (35)
Total	32	25	21	78 (100)

In the entire population of 79 patients (data on diastolic dysfunction were missing at follow-up for one patient), 95% of patients with grade I diastolic dysfunction maintain unchanged diastolic function, 31% of patients with dysfunction Grade II diastolic passes to grade I, 37% of patients with grade III dysfunction improve diastolic function.

Legend: Bold: unchanged, green: improved, orange: worsened. Fisher's exact p value test=7.10×10^{-12}.

Table S5. Change in diastolic dysfunction at follow-up in non-ischemic patients.

Non-ischemic	**Diastolic dysfunction at follow-up**			
**Diastolic dysfunction at baseline**	1	2	3	Total
1	**11 (58)**	0 (0)	0 (0)	11 (31)
2	7 (37)	**10 (100)**	1 (14)	18 (50)
3	1 (5)	0 (0)	**6 (86)**	7 (19)
Total	19	10	7	36 (100)

In the subgroup of 36 non-ischemic patients (one patient data is missing), 100% of patients with grade I diastolic dysfunction maintain diastolic function unchanged, 38.8% of patients with grade II diastolic dysfunction pass to grade I, 14% of patients with grade III dysfunction improve diastolic function (Table 11). Legend: Bold: unchanged, green: improved, orange: worsened. Fisher's exact p value test=2.9x10^{-7}.

Table S6. Change in diastolic dysfunction at follow-up in ischemic patients.

Ischemic	**Diastolic dysfunction at follow-up**			
**Diastolic dysfunction at baseline**	1	2	3	Total
1	**10 (77)**	1 (7)	0 (0)	11 (26)
2	2 (15)	**6 (40)**	3 (21)	11 (26)
3	1 (8)	8 (53)	**11 (79)**	19 (48)
Total	13	15	14	42 (100)

In the subgroup of 42 ischemic patients, 90.9% of patients with grade I diastolic dysfunction maintain unchanged diastolic function, 18% of patients with dysfunction Grade II diastolic passes to grade I, 47% of patients with grade III dysfunction improve diastolic function. Legend: Bold: unchanged, green: improved, orange: worsened. Fisher's exact p value test=8.8x10^{-6}.

Table S7. Differences in clinical parameters between follow-up and baseline.

**Follow-up—baseline**	**Non ischemic (N=37)**	**Ischemic (N=42)**	**All (N=79)**	**P value non ischemic (N=37)**	**P value ischemic (N=42)**	**P value All (N=79)**
**Systolic pressure (mmHg)**	-6 (15)	-9 (14)	-7 (15)	**0.0059**	**0.0005**	**9.23x10^{-6}**
**GFR (ml/min/1.73m^2)**	-1 (12)	-4 (13)	-3 (12)	0.6196	**0.0189**	**0.0439**
**NT-proBNP (pg/mL)**	-571 (-3922; 64)	-251 (-2180; 489)	-538 (-3156; 279)	**0.0236**	0.2129	**0.0159**
**Furosemide dosage (mg)**	-3 (29)	-15 (46)	-10 (39)	0.4108	**0.0180**	**0.0221**

Table S8. Predictive factors of reverse remodeling. *Beta expresses the percentage change of delta EDV from ischemic to non-ischemic patients. The beta coefficient expresses the increase (if> 0) or decrease (if <0) estimated for the delta EDV%, passing from the group of ischemic patients (reference category for comparison) to non-ischemic. The variance explained by the model (R2) indicates what percentage of variability can be predicted by the covariates (or predictors). N = sample size actually used in the model SE = standard error CI = confidence interval.*

Outcome: *reverse remodeling*	N	Beta (SE)	95% CI	P value	Variance explained by the model (R^2)
**1. Diagnosis (ischemic/non-ischemic)**	79	-4.26 (3.60)	-11.42; 2.90	0.240	1.80%
**2. Diagnosis (age, sex)**	79	-3.32 (3.83)	-10.95; 4.31	0.389	3.28%
**3. Diagnosis (age, gender, time since HF diagnosis, baseline NT-proBNP, baseline furosemide dose, baseline degree of diastolic dysfunction, baseline LAV, follow-up time, final SV dose)**	68	-7.77 (4.30)	-16.39; 0.84	0.076	13.79%

Supplementary Figures

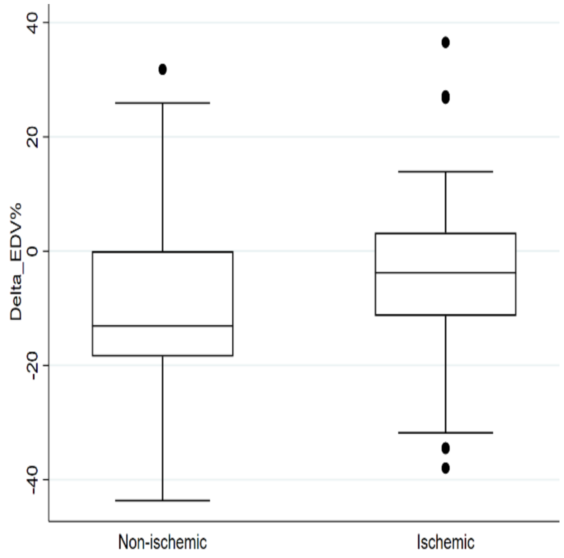

Delta EDV%	Range	Average (SD)	Median (IQR)
**Non ischemic**	-43.67; 31.82	-8.67 (15.72)	-13.08 (-18.44; 0)
**Ischemic**	-37.96; 36.54	-4.41 (16.16)	-3.76 (-11.35; 3.25)

SD= standard deviation; IQR= interquartile range

Figure S1. Graphical representation and descriptive statistics relating to the delta EDV%.

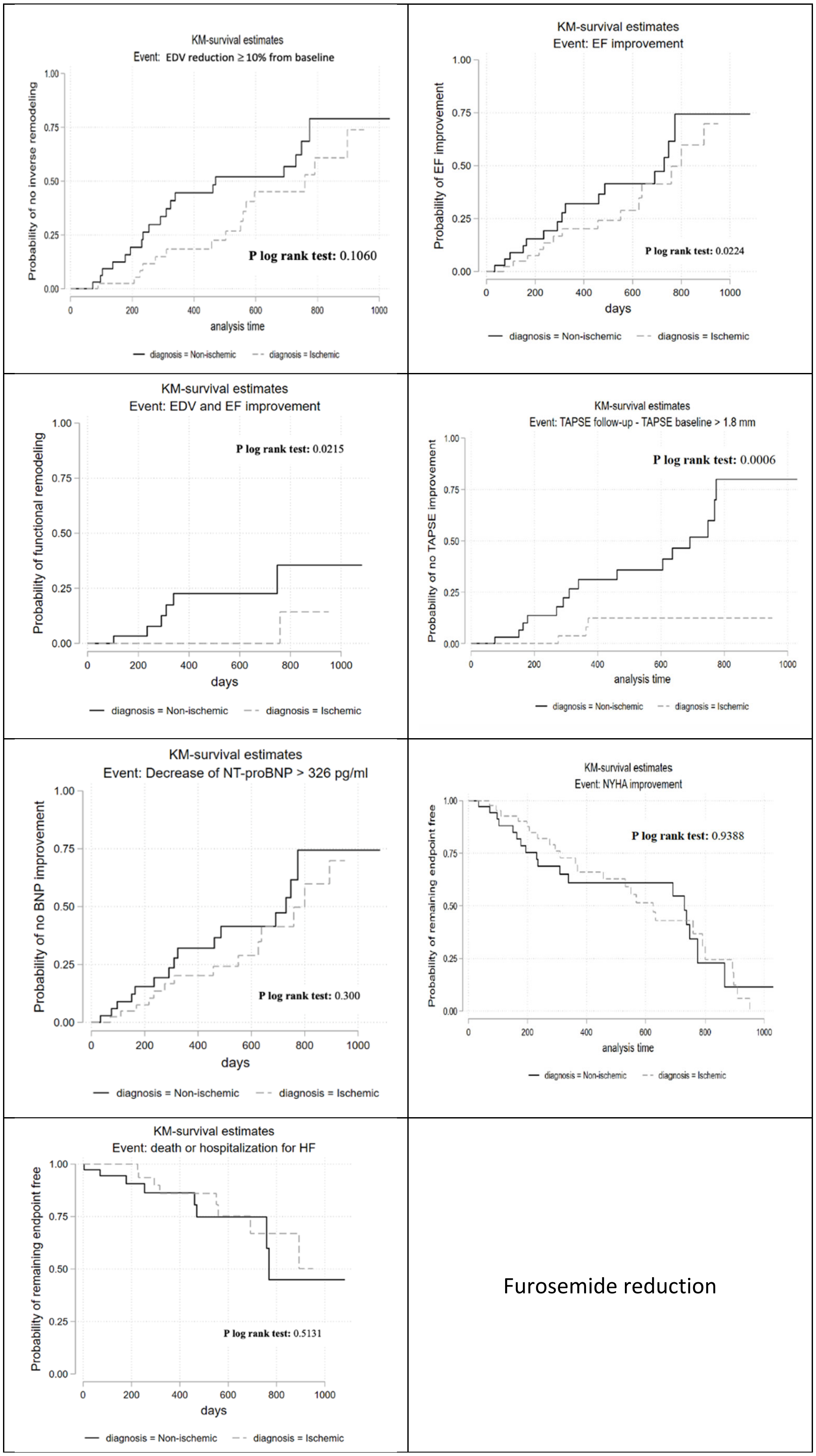

Figure S2. Kaplan Meier survival analysis.
